# Conformational stability of digestion-resistant peptides of peanut conglutins reveals the molecular basis of their allergenicity

**DOI:** 10.1038/srep29249

**Published:** 2016-07-05

**Authors:** Danijela Apostolovic, Dragana Stanic-Vucinic, Harmen H. J. de Jongh, Govardus A. H. de Jong, Jelena Mihailovic, Jelena Radosavljevic, Milica Radibratovic, Julie A. Nordlee, Joseph L. Baumert, Milos Milcic, Steve L. Taylor, Nuria Garrido Clua, Tanja Cirkovic Velickovic, Stef J. Koppelman

**Affiliations:** 1 Center of Excellence for Molecular Food Sciences, University of Belgrade - Faculty of Chemistry, Studentski trg 16, 11000 Belgrade, Serbia; 2 TI Food and Nutrition, P.O. Box 557, 6700 AN Wageningen, the Netherlands; 3 TNO, Utrechtseweg 48, 3704 HE, Zeist, the Netherlands; 4 Institute of Chemistry, Technology and Metallurgy - Center of Chemistry, Njegoseva 12, Belgrade, Serbia; 5 Food Allergy Research and Resource Program, University of Nebraska, 279 Food Innovation Center, Lincoln, Nebraska 68588-6207, USA

## Abstract

Conglutins represent the major peanut allergens and are renowned for their resistance to gastro-intestinal digestion. Our aim was to characterize the digestion-resistant peptides (DRPs) of conglutins by biochemical and biophysical methods followed by a molecular dynamics simulation in order to better understand the molecular basis of food protein allergenicity. We have mapped proteolysis sites at the N- and C-termini and at a limited internal segment, while other potential proteolysis sites remained unaffected. Molecular dynamics simulation showed that proteolysis only occurred in the vibrant regions of the proteins. DRPs appeared to be conformationally stable as intact conglutins. Also, the overall secondary structure and IgE-binding potency of DRPs was comparable to that of intact conglutins. The stability of conglutins toward gastro-intestinal digestion, combined with the conformational stability of the resulting DRPs provide conditions for optimal exposure to the intestinal immune system, providing an explanation for the extraordinary allergenicity of peanut conglutins.

Peanut allergens have been extensively studied for decades for their biological and immunologic properties^1^. Peanut conglutins, Ara h 2 and Ara h 6, represent the allergens recognized by the majority of peanut allergic patients^2^. These allergens are far more potent than other peanut allergens like Ara h 1 and Ara h 3, as was shown by effector cell activation and *in vivo* in human skin^2^,^3^. Ara h 2 and Ara h 6 together are responsible for 80–90% of the allergenic activity of the total peanut extract as determined by activation of basophils using a rat basophil leukemia cell line assay with IgE from peanut allergic patients^4^,^5^,^6^,^7^. Ara h 2 was described as pre-eminent in importance because it was identified as a predictor of clinical reactivity to peanut^8^,^9^,^10^,^11^. Recently, it was shown that Ara h 6 is good predictor of clinical reactivity too, and complements Ara h 2 for IgE reactivity^10^,^12^.

Ara h 2 and Ara h 6 are 2S albumins with four tightly coiled helical structures that form a heat- and protease-stable core^13^. They are isoforms of each other with 59% sequence homology^14^. Ara h 2 exists in two isoforms, Ara h 2.02 and Ara h 2.01^15^,^16^, with reported masses of 18032 Da and 16341 Da, respectively, determined by mass spectrometry^17^. In comparison with Ara h 2.01, Ara h 2.02 has an insertion of 12 amino acids, starting at residue 54 containing the linear IgE-binding epitope, DPYSPS^18^. For Ara h 2, site-specific proline hydroxylation has been reported earlier, together with the mapping of disulphide linkages^19^. Ara h 6 has a determined mass of 14835 Da^17^,^20^. It has been shown that conglutin isoforms from peanut are very resistant to gastro-intestinal (GI) digestion, even after heat treatment^13^,^20^,^21^,^22^,^23^.

Peanut allergens have been detected in breast milk by immunoassays, suggesting that at least some of the immunoreactive domains of these peanut allergens remain intact during uptake by intestinal epithelium and entry into the circulatory system^24^,^25^. Earlier studies showed that digestion of conglutins results in digestion-resistant peptides (DRPs) with approximate masses of 10 kDa^13^,^20^, regardless whether gastric or intestinal proteases were used^22^,^23^,^26^. The digestion-resistant peptide from Ara h 2 (DRP-Ara h 2) could be detected in serum from health individuals for up to 24 hours after ingestion, while the presence of intact peanut protein could not be demonstrated^27^. Transfusion-related anaphylaxis occurred due to the passive transfer of peanut allergen present in donor blood to a peanut-allergic recipient, indicating *in vivo* functionality of peanut allergen circulating in blood after ingestion^28^. Although the main part of the *in vivo* studies was done with an immunoassay directed against Ara h 2, the exact nature of the peanut allergen found in the circulation is not known. Still there is limited information on primary sequence of the DRPs.

The aim of this study is to identify the digestion resistant peptides for each of the naturally occurring conglutins Ara h 2.01, Ara h 2.02, and Ara h 6 in order to understand the epitope diversity of these major peanut allergens. Furthermore, we applied a molecular dynamics simulation to understand the cleavage sites of the protease and the effects of the digestion on the overall conformational stability and conformational IgE-binding epitopes of these peanut allergens.

## Results

### Trypsin digestion gives stable, digestion-resistant peptides (DRPs) for all conglutin isoforms

Peanut conglutin isoforms, Ara h 2.02, Ara h 2.01 and Ara h 6, were digested by incubation with trypsin. Supplementary Fig. S1 shows the time course of the digestion as visualized by Sodium dodecyl sulfate polyacrylamide gel electrophoresis (SDS-PAGE) under reducing conditions. The peptides originating from the digestion remain stable up to the 180 minutes endpoint of our study for all of the conglutin isoforms. DRPs of the Ara h 2.02 isoform consist of two groups, with apparent molecular masses of approximately 12 kDa, and 10 kDa. DRPs from Ara h 2.01 exhibit an apparent molecular weight of approximately 10 kDa and careful examination of this area reveals two co-migrating bands. DRPs from Ara h 6 migrate as approximately 9 kDa and 5 kDa bands. For further characterization, DRPs prepared by digestion for 90 minutes were used. Under physiological conditions using pepsin at low pH followed by trypsin/chymotrypsin at neutral pH, peanut conglutins are resistant to proteolysis as well. The resulting DRPs have similar molecular weights as the ones analyzed in our study, and can still bind IgE (data not shown), in accordance to other studies^13^,^22^.

### Identification of sequences of DRPs from peanut conglutin isoforms

**Figure 1:**
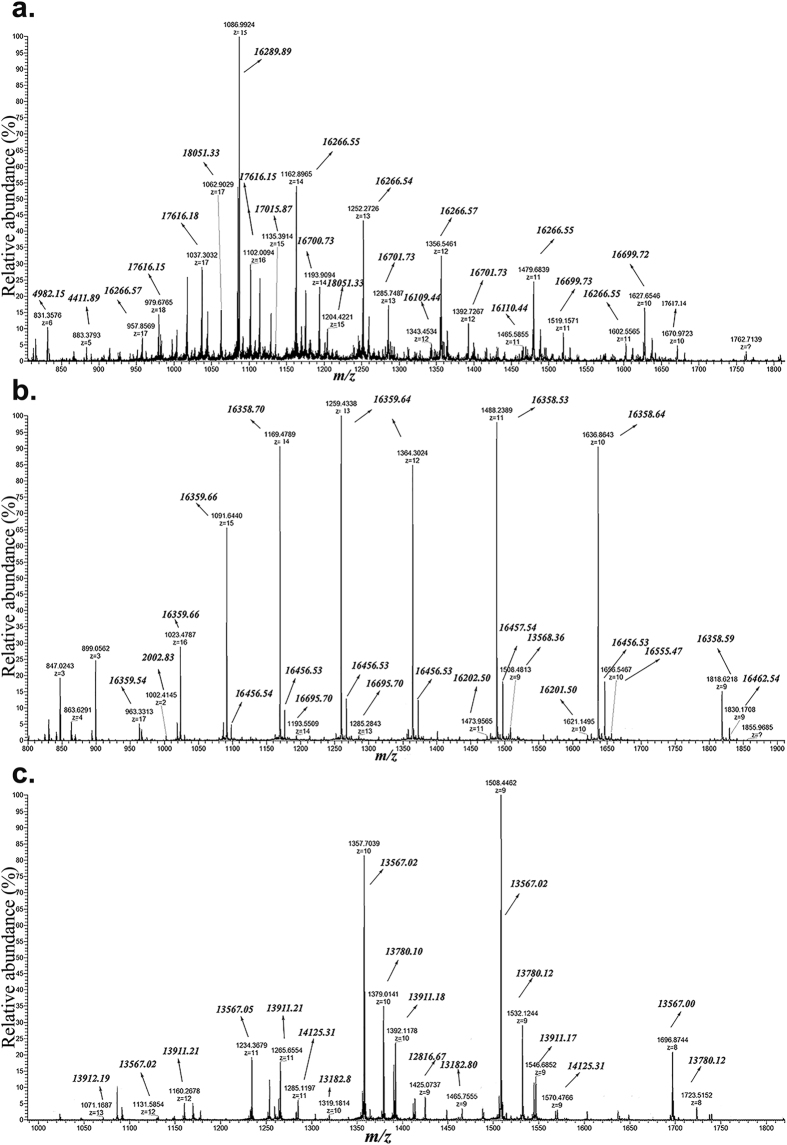
ESI-MS spectra of non-reduced DRPs. Non-reduced DRPs were subjected to ESI-MS to determine masses of individual peptides. Peptides may be linked by disulphide bonds. Panel a: DRPs of Ara h 2.02; Panel b: DRPs of Ara h 2.01; Panel c: DRPs of Ara h 6.

**Figure 2:**
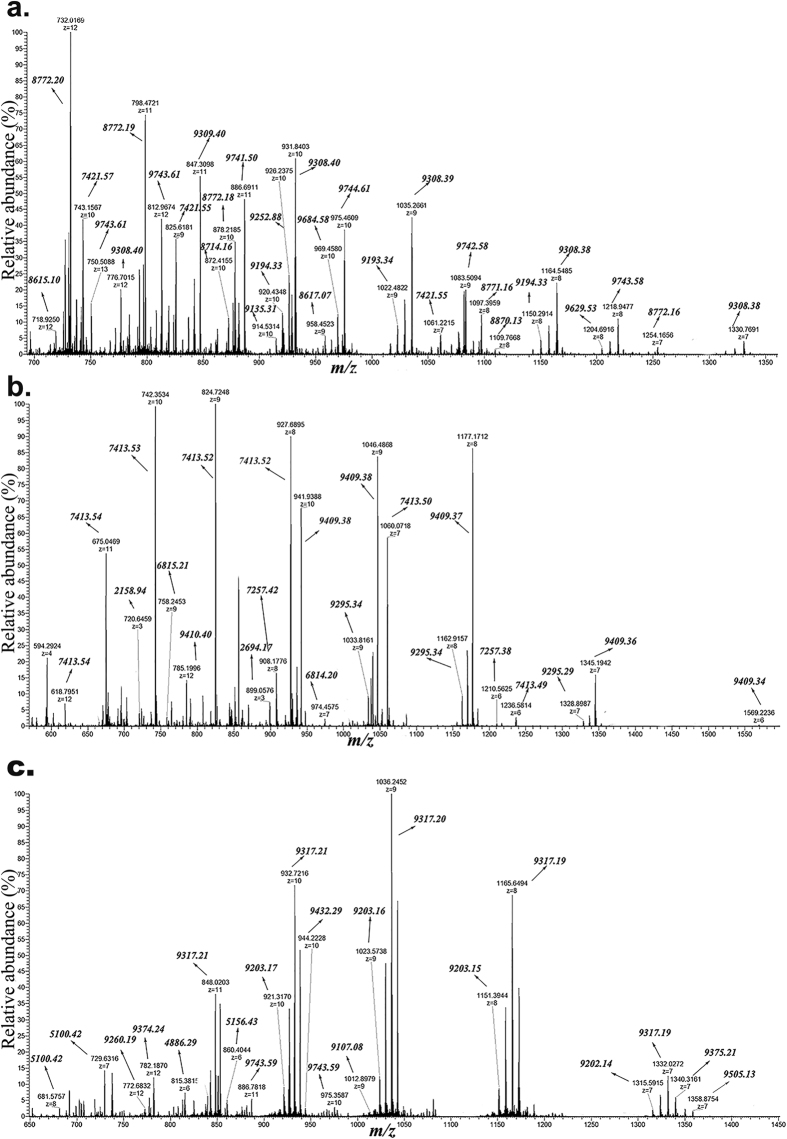
ESI-MS spectra of reduced and alkylated DRPs. Reduced and alkylatd DRPs were subjected to ESI-MS to determine masses of individual peptides. Panel a: DRPs of Ara h 2.02; Panel b: DRPs of Ara h 2.01; Panel c: DRPs of Ara h 6.

High-resolution mass spectra were recorded for non-reduced and reduced- and alkylated DRPs from Ara h 2 isoforms, Ara h 2.02 and Ara h 2.01, and Ara h 6. ESI-MS spectra of non-reduced and reduced- and alkylated DRPs are shown in Figs 1 and 2, respectively. For non-reduced DRPs, a wide range of m/z ions with higher charge states (from +8 to +17) is observed, which represent masses with molecular masses >13 kDa. The mass spectra for these non-reduced DRPs also contain m/z ions with lower charge states (from +2 to +6), representing the masses <5 kDa. Reduced and alkylated DRPs give a charge state range from +4 to +12, representing the masses from 2.2–10 kDa. In the spectra of the reduced and alkylated DRPs, it can be observed that one charge state ion gives several m/z masses, deviating 57 Da from each other. This relates to partial alkylation of cysteines. The Supplementary Table S1 summarizes the masses found for the DRPs, both from non-reduced and reduced and alkylated form. Trypsinolysis of Ara h 2.02 and 2.01 led to DRPs of molecular masses from 16.2–17.6 kDa, as well as to some small peptides from 2–4 kDa (see Supplementary Table S1). Ara h 6 showed DRPs of molecular masses from 13.5–14.1 kDa. Taking into account the known post-translational modification, post-translational processing and combining of the masses of intact and reduced and alkylated DRP using the mapping of disulphide bonds^19^, the experimental masses were linked to theoretical masses (see Supplementary Table S1).

**Figure 3:**
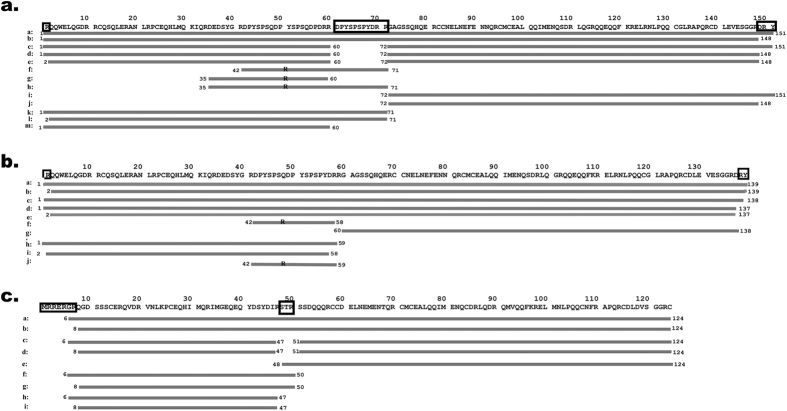
Sequences of the DRPs as determined by nanoLC-MS. Using the sequence of the intact conglutins (top line of each panel), the identified peptides are shown by subsequent lines (indicated with letters). Each line represents a unique peptide found. Lines marked with ‘R’ represent peptides that are released from the protein core upon digestion. Panel a: Sequences of the DRPs from Ara h 2.02 protein species; Panel b: Sequences of the DRPs from Ara h 2.01 protein species; Panel c: Sequences of the DRPs from Ara h 6 protein species.

Two dimensional electrophoresis (2-DE) profiles of DRPs from individual conglutin isoforms (see Supplementary Fig. S2) were used to assign spots, where different pI values of DRPs provided additional directions for peptide verification. Figure 3 provides an overview of the peptide sequences found in the DRPs of the three peanut conglutins. After digestion we have detected proteins with one peptide bond hydrolysed on the Arg_59/71_ for Ara h 2 and Arg_50_ for Ara h 6 (Fig. 3. Panel a - Ara h 2.02, peptides a, and b; Panel b - Ara h 2.01, peptides b, c, d, and e; Panel c - Ara h 6, peptides a, and b), which after reduction yields two peptides with molecular weights from 7.2–9.7 kDa for Ara h 2 and 4.8-9.4 kDa for Ara h 6. In the intact DRPs from Ara h 2, a C-terminal proteolytic processing of the Y/RY fragment was observed^29^, but also cleavage at the trypsin cleavage site Arg_149/137_-Asp_150/138_. Additionally, peptides with deleted internal short segments (S_48_TR_50_ in Ara h 6 and D_61_PYSPSPYDRR_71_ in Ara h 2.02) were detected. In DRPs from Ara h 2, internal segments were detected starting at Asp_35_ or Asp_42_ (Fig. 3. Panel a – Ara h 2.02 peptides f, g and h, Panel b – Ara h 2.01 peptide f). These short peptides do not contain cysteine residues and do not remain associated to the stable protein core. The N-terminus of the Ara h 6 after digestion showed some diversity of N-terminus (proteolysis at Arg_5_ or Arg_7_, Fig. 3. Panel c – Ara h 6 peptides a–d). For all three conglutins, the most digestion-susceptible parts are the N- and C-terminus, and to a lesser extent a limited internal part. It is evident that in all three conglutin isoforms, the main internal site of trypsin attack is structurally identical (R_59_|R_60_|D_61_PYSPSPYDRR_71_|G_72_ in Ara h 2.02, R_58_|R_59_|G_60_ in Ara h 2.01 and R_47_|S_48_TR_50_|S_51_ in Ara h 6), located in the loop that bears no defined local structure. Supplementary Fig. S3 contains sequence alignment of the conglutin isoforms with assignment of secondary structure elements and 2D topology diagram showing the disulphide bridges.

### The secondary structure of the peanut conglutin isoforms is not affected by digestion and molecular dynamic simulation reveals conformationally stable structures of DRPs

**Figure 4:**
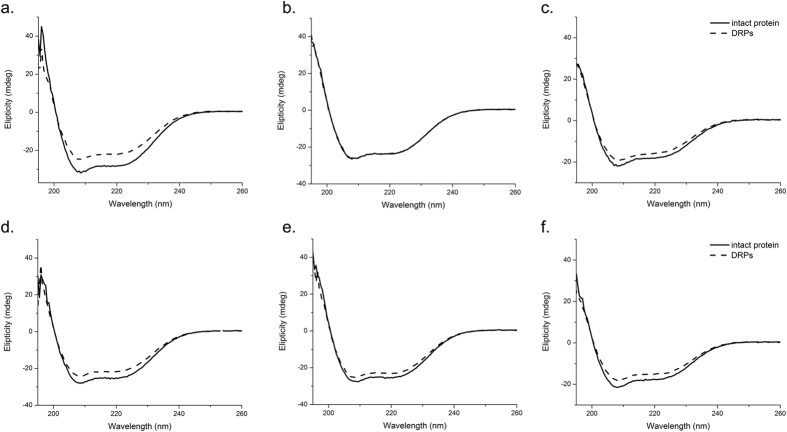
Far UV CD spectra of DRPs versus native conglutin isoforms. Native conglutins and their DRPs were analysed by far UV CD spectroscopy to investigate secondary structure content, both at neutral pH and low pH to mimic gastric and duodenal conditions. Solid lines represent native peanut conglutins; dotted lines represent DRPs from the respective conglutins. Top panels (a,b,c) at pH = 8.0, lower panels (d,e,f) at pH=1.2. Left panels (a,d): Ara h 2.02; Middle panels (b,e): Ara h 2.01; Right panels (c,f): Ara h 6.

Far UV Circular dichroism (CD) spectra were acquired at pH 8 and pH 1.2, at 37 °C, in order to gain better understanding of the structural properties of DRPs of Ara h 2.02, Ara h 2.01 and Ara h 6. The spectra of the three native isoforms show a strong positive ellipticity from 200 to 190 nm, typically indicating the presence of α-helical structures (line plots in Fig. 4). The shape of the far UV CD spectra of DRPs is similar to that of native proteins (dashes plots in Fig. 4). The zero-crossing, i.e. the wavelength at 0 mdeg ellipticity, is the same for DRPs and native isoforms, confirming that no notable change in secondary structure occurred as a result of degradation of the intact protein into smaller fragments. Native conglutins and their DRPs have highly comparable far UV CD spectra at neutral pH and low pH, indicating that no denaturation occurs in the stomach.

**Figure 5:**
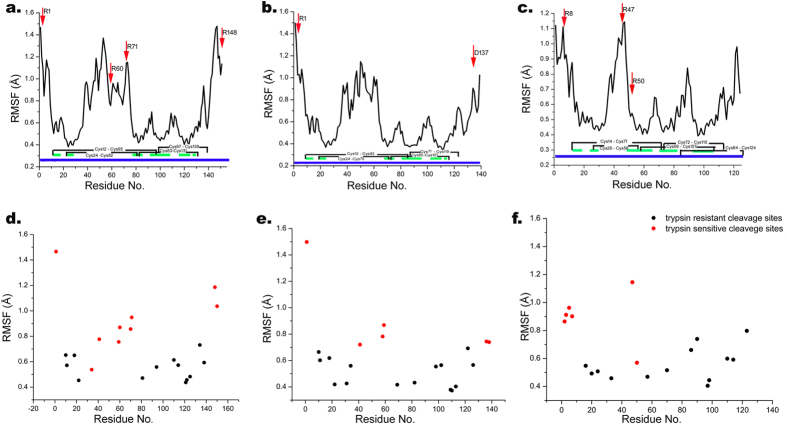
RMSF of intact conglutin isoforms with trypsin affected sites in DRPs (d,e,f). Top panels (a,b,c): Intact conglutins, lower panels (d,e,f): Trypsin affected sites in DRPs. Left panels (a,d): Ara h 2.02; Middle panels (b,e): Ara h 2.01; Right panels (c,f): Ara h 6.Arrows indicates place which are affected with trypsin; Blue line represent the protein sequence; Green lines represents the parts of protein sequences which contains α-helix as secondary structure; C represent cysteine which is involved in formation of disulphide bond.

To analyse the consequences of the proteolytic action for the three isoforms, a molecular dynamics simulation was performed on both the intact and digested proteins for 500 ns for Ara h 2 and 800 ns for Ara h 6. According to backbone root mean square fluctuations (RMSF), it can be noticed that both isoforms of Ara h 2 have three regions with high mobility: the flanking N-terminal part up to the first Cys, the C-terminal part from the last Cys residue to the end of the sequence, and a region which is almost the entire unstructured loop between second and third Cys residue (Fig. 5a,b). The mobility of Ara h 6 regions is similar, except that the C-terminal part is not mobile due to a disulphide located at the C-terminus of the protein (Fig. 5c). Trypsin cleavage sites are marked with an arrow in Fig. 5 for the DRPs and these are considered for further molecular dynamics analysis. Furthermore, Fig. 5d–f show trypsin-sensitive cleavage sites for all DRPs identified in this study (Fig. 3). It can be observed that RMSF values of almost all trypsin-sensitive sites found in this study are higher than 0.60 Å, (Fig. 5d–f), while trypsin resistant sites are virtually all showing RMSF values smaller than 0.70 Å. In fact, the positive predictive value for the susceptibility of target cut sites in peanut conglutins to trypsinolysis is 83%, for resistance to proteolytic action is 87%, if the cut RMSF value was set at 0.65 Å.

**Figure 6:**
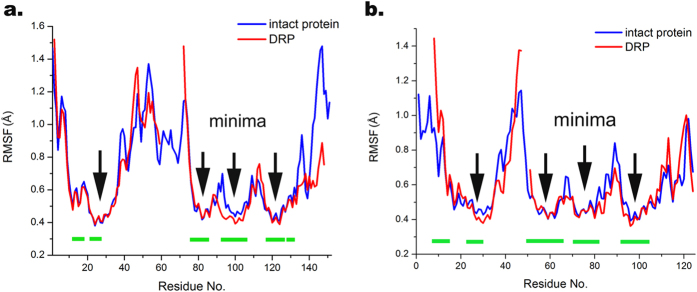
RMSF variations of intact conglutin isoforms and DRPs. Panel a: Comparison of Ara h 2.02 and its DRP (Fig. 3a, peptide d); Panel b: Comparison of Ara h 6 and its DRP (Fig. 3c, peptide d). Arrows represent the minima of the RMSF which position is located in the α-helical region of the protein. Green lines represent the parts of protein sequences, which contain α-helix as secondary structure.

**Figure 7:**
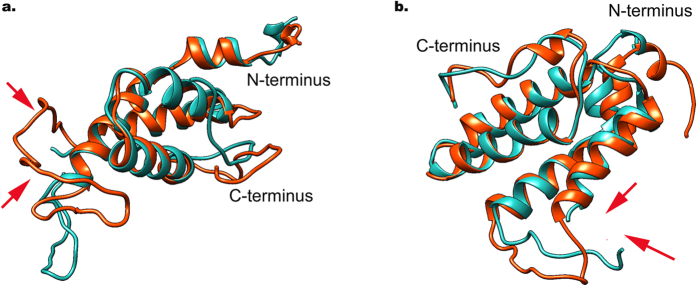
3D structure of DRP overlapped with intact protein. Panel a: Ara h 2.02; Panel b: Ara h 6. The structure in red represents conformation of intact proteins and the structure in blue represent DRP (peptide d from Fig. 3a,c). Arrows show cleavage sites, indicating that the non-structured parts are affected.

Four local RMSF minima could be seen with <0.5 Å, corresponding to the α-helical regions (Fig. 6), i.e. the highly structured protein core. According to Fig. 6, which represents overlapped RMSF values of intact protein with its corresponding DRPs, local mobility of DRPs demonstrates a similar pattern, including α-helical regions with RMSF minima. Figure 7 represents the 3D structure of overlapped intact protein with its corresponding DRP. In the DRPs, only a small conformational change could be observed, for Ara h 2 in the non-structured loop and the C-terminal region, and for Ara h 6 deviations are observed in the non-structured loop and the N-terminal region, while α-helical regions remained in their helical structure without any helical movement and rearrangement for all conglutin isoforms (Fig. 7), and even become slightly less dynamic. These results indicate that proteolysis does not substantially change the conformational stability of the proteins.

The root mean square deviation (RMSD) and radius of gyration of the DRPs was calculated by evaluating the spatial deviations in structure. This analysis further confirmed the compactness of DRPs (see Supplementary Figs S4 and S5). During the entire simulation time, DRPs of both Ara h 2 and Ara h 6 show a lower RMSD profile, suggesting that they are more stable than the corresponding intact proteins.

Radius of gyration (Rg) values reveal a lower plot of DRPs during the whole simulation time and imply that DRPs have noticeably more compact conformation in comparison to intact protein. The more compact structure of DRPs than corresponding intact proteins (with more pronounced effect in the case of Ara h 2), as well as higher compactness of Ara h 6 than Ara h 2, is in accordance to changes in protein secondary structure elements during the simulation (see Supplementary Fig. S6) e.g. noticeably higher content of helixes in DRPs than in intact Ara h 2 proteins and higher content of helixes in Ara h 6 than in Ara h 2. This can be explained by the loss of non-structured amino acid sequences resulting in a higher fraction of structured elements in the digestion products.

### Digestion-resistant peptides exhibit IgE-binding properties like native conglutin isoforms

**Figure 8:**
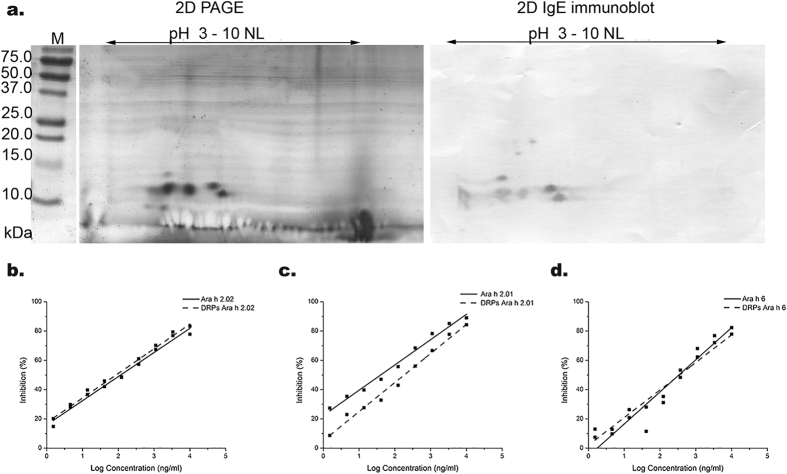
IgE-binding properties of DRPs in comparison with native peanut conglutin isoforms. Panel a: 2-D electrophoresis (left) and 2-D electrophoresis wit IgE Immunoblot of DRPs from Ara h 2/ h 6 mixture. M: Molecular markers; Panel b-d: IgE-binding potency as determined by IgE-ELISA of digestion-resistant peptides (dashed lines) and native conglutin (solid lines). A typical example is shown (Panel b: Ara h 2.02; Panel c: Ara h 2.01; Panel d: Ara h 6). IgE binding potencies of DRPs are similar to those of intact conglutins.

The IgE-binding of DRPs from Ara h 2 and Ara h 6 was assessed by 2D gel electrophoresis combined with immunoblotting. Figure 8a shows that the three isoforms gave rise to IgE-binding spots. DRPs of Ara h 6 have more acidic pIs than DRPs from Ara h 2 isoforms. IgE-immunoblotting also reveals additional spots with higher molecular weights. This represents a small fraction of intact Ara h 6, which remains after 90 min of digestion, as noted on SDS-PAGE and 2-DE. Spots that did not bind IgE (at <10 kDa and with acidic pI) appeared to originate from Ara h 6 (Fig. 8a and Supplementary Fig. S2).

Additionally the IgE-binding potency of DRPs compared to that of intact peanut conglutin was assessed on a quantitative level by IgE-ELISA. Figure 8 (panels b–d) shows the inhibition plots of Ara h 2.02, Ara h 2.01, and Ara h 6 and their DRPs. For Ara h 2.02 and Ara h 6, the inhibition plots of DRPs are virtually overlapping with those of the intact proteins. For Ara h 2.01, there is a small shift to higher concentration, suggesting a slightly lower IgE-binding potency. From three independent experiments, the mean IC_50_ values (the concentration needed to inhibit 50% of the signal) were obtained and the fold increase in IC_50_ value between DRPs and intact proteins was determined. For Ara h 2.02, Ara h 2.01, and Ara h 6, this fold increase was 0.8 (±0.4), 1.4 (±1.4), and 1.5 (±0.6), respectively. As these numbers are close to 1, it can be concluded that the IgE-binding potency of all three peanut conglutin isoforms is not affected by trypsinolysis.

## Discussion

Peanut conglutins Ara h 2 and Ara h 6 are known to be resistant to pepsin proteolysis under gastric conditions^20^,^23^,^30^ and trypsin/chymotrypsin proteolysis under intestinal conditions^13^,^20^,^26^. Digestion with either of both proteases yields DRPs with apparent molecular weights similar to that of the native protein^13^,^23^, indicating that the actual susceptible cleavage sites for digestive proteases are determined by factors other than by protease specificity alone.

In this study, we obtained DRPs from conglutin isoforms with the molecular weights ranging from 5 to 10 kDa, that are held together by disulphide bonds and form a tight digestion-resistant core with a mass slightly lower than native conglutins, in line with earlier publications^13^,^20^,^21^,^26^. We have elucidated the complete sequences of DRPs and show for all three conglutin isoforms that except for the cleavage of the N-terminal part from the C-terminal part, heterogeneity in digestion occurs. This is in line with the 3D model for Ara h 6 and super-positioning of Ara h 2 onto it^26^, which confirms that the region with observed cleavage heterogeneity matches with non-structured parts. Furthermore, the size of the peptides derived corresponds with the size of the non-structured segments of the different conglutin isoforms. In contrast to Ara h 2, the C-terminus of Ara h 6 is not processed by trypsin due to cysteine residues involved in a disulphide bond (Cys_84_-Cys_124_) that limits the accessibility of Arg_123_. Regardless of the observed heterogeneity in DRPs, it is clear that most potential cleavage sites remain unperturbed even upon prolonged digestion. Most of these potential protease cleavage sites appear to reside in close proximity of the cysteine-residues. It is thus likely that such sites are inaccessible to the active site of trypsin due to steric hindrance by disulphide bonds, and their location in the compact tightly coiled core, CXnCXnCCXnCXCCXnC, typical for 2S albumins^31^. Indeed, 2S albumins from Brazil nut^32^, sesame seed^33^, sunflower^34^, cashew^35^, and hazelnut^36^ all exhibit a poor digestibility under gastro-intestinal conditions.

Our far UV CD data confirm that the secondary structure of the proteins is stable at both neutral and acidic pH and preserved after prolonged digestion with trypsin. To further analyse the conformation and stability of the fold of identified DRPs, a molecular dynamics simulation was performed on the modeled 3D structures of DRPs. The molecular dynamics simulation of DRPs demonstrated that the conformationally vibrant regions, such as the internal non-structured loop in Ara h 6 and Ara h 2.02, as well as the termini of the proteins, represent the main proteolysis target sites. In contrast, the most rigid regions are those in the immediate vicinity of Cys residues that form disulfide bridges, as well as regions in α-helical conformation. Although these regions contain numerous cleavage sites for trypsin they remain resistant for a prolonged time.

Trimming-off the non-structured parts of the conglutins by proteolysis leads to relatively more structured and more stable DRPs. This effect is greater for Ara h 2 than for Ara h 6, because Ara h 2 has a higher content of non-structured amino acid sequences. It is interesting to note that the difference between intact conglutin and DRPs is the smallest for Ara h 6, due to a smaller content of non-structured amino acid sequences and the presence of one extra disulphide bond compared to Ara h 2. One could suggest that the DRPs of the different conglutin isoforms are more similar than their native counterparts, which may explain functional similarities while clear differences on the protein structural level are observed. Although for 2S albumins from other legumes, seeds, and nuts the cleavage sites are not fully known, it can be suggested that proteolysis of very diverse 2S albumins may result in DRPs with highly comparable structures because non-structural parts are cleaved-off.

It was shown previously that digested Ara h 2^13^ and Ara h 6^21^ were still able to bind IgE. In this study, we show that DRPs of both conglutins have the same IgE-binding potency as their intact counterparts. The retained IgE-binding potency, combined with the preserved protein structure, supports the hypothesis that IgE-binding to conglutins is primarily dependent on conformational epitopes^17^,^37^ and suggests that linear IgE epitopes in the non-structured areas, as identified by Stanley^38^, are less relevant.

Apart from the IgE-binding aspects, the digestive stability of allergens is also associated with higher immunogenicity, i.e. the potential to induce an immune response on a cellular level^39^. Recently it was also shown that conformational stability of allergens is a determinant for immunogenicity^40^. Our data show that peanut conglutins Ara h 2 and Ara h 6 fulfill both criteria; they confer stability toward digestion, combined with a high structural stability of the resulting DRPs, and may thus provide an explanation for extraordinary allergenicity of peanut conglutins.

This work identified and elucidated the structures of DRPs of naturally occurring isoforms of peanut conglutins. We have shown that proteolysis targets reside in highly vibrant parts of the proteins. The DRPs resemble their native counterparts in terms of folding, conformational stability, and IgE-binding. These stability attributes allow DRPs to enter the small intestine to trigger the gut immune system, and to be taken up in circulation eliciting systemic allergic reactions.

We propose that conformational stability and resistance towards digestion may be the structural basis for the allergenicity of peanut conglutins Ara h 2 and Ara h 6, and other similarly structured 2S albumins.

## Methods

### Trypsin digestion of peanut conglutin isoforms

Trypsin immobilized onto magnetic beads was used (ClonTech Laboratories Inc., Mountain View, CA, USA, product number 635646). The activity of trypsin was determined using the substrate alpha-benzoyl-L-arginine ethyl ester (BAEE, Sigma-Aldrich Corp., B4500, St. Louis, MO, USA), according to the instructions of the substrate manufacturer. The activity of the immobilized trypsin suspension (specification of the manufacturer >150 U/ml) was 218 U/ml. A comparison of activity with soluble trypsin was made in order to express the trypsin concentration of the immobilized trypsin in μg/ml. Thereto, trypsin (Sigma-Aldrich, product number T-1426, with a claimed specific activity of 10,000 U/mg), was assayed for specific activity of 9,560 U/mg. The activity of the immobilized trypsin of 218 U/ml corresponds to 22.8 μg/ml of trypsin in the stock suspension. Purified Ara h 2.01, Ara h 2.02, and Ara h 6, as well as a mixture of Ara h 2 and Ara h 6, were diluted to 0.5 mg/ml with 6.5 mM Tris-HCl pH 8 with the final volume of 1 ml. Thirty μl of a suspension of trypsin immobilized onto magnetic beads was added to the protein solution, corresponding to a final trypsin concentration of 0.68 μg/ml. Samples were incubated at 37 °C in an incubator shaker (1400 rpm). At different time points, aliquots were taken, beads separated from the solution using a magnet outside the vial, and the samples were then analysed on SDS-PAGE under reducing conditions. 2 μg of digested material were run on 12% Bis-Tris gels with MES buffer using conditions set out by the manufacturer (Bio Rad, Hercules, CA, USA). DRPs obtained after 90 minutes of trypsin digestion were used for further analysis.

### Mass spectrometry analysis

DRPs were analysed on EASY nanoLC II system coupled with LTQ Orbitrap XL (Thermo Fisher Scientific Inc., Waltham, Massachusetts, USA), previously calibrated with the ProteoMass^™^ LTQ/FT-Hybrid ESI Positive Mode Cal Mix (MSCAL5 SUPELCO, Sigma-Aldrich) calibration set. Samples were also analysed under reducing and alkylating condition. Reduction and alkylation of the DRPs was done as previously described for peanut conglutins^17^. Two microliters (concentration of 50 μg/ml) of each sample was injected onto the trapping column (EasyColumn C18, 2 cm length, ID 100 μm, 5 μm particle size) and separation was performed on an Easy spray PepMap C18, (length 15 cm, ID 75 μm, particle size 3 μm) as described^17^. Spray was generated with a stainless steel emitter, with tip voltage set at 2.35 kV, capillary voltage 6 V and capillary temperature of 275 °C. A high-resolution full Fourier-Transform Mass Spectrometry (FTMS) profile spectrum (scan range 300–3000 m/z, resolving power 60 000, 1 microscan) was acquired using Xcalibur (version 2.1) software (Thermo Fisher Scientific) with the precursor mass tolerance of 10 ppm. The experiments were done in duplicate.

### Determination of digestion-resistant peptides

Prediction of the sequences of the DRPs was based according to the exact masses obtained by MS and exact masses of the native isoforms, using the peptide calculator tool (http://pepcalc.com/). For theoretical mass calculation in the peptide calculator, amino acid sequences for conglutin isoforms (Uniprot Q6PSU2-1, Q6PSU2-4 and Q647G9) were used, including the sequence conflicts. To take into account hydroxylation of proline as a posttranslational modification, partial reduction and alkylation, a mass addition of 16 Da for each Pro_46_, Pro_53_ and Pro_65_, and 57 Da for each alkylated Cys, were used^19^. For locating potential trypsin-cleavage sites, the ExPaSy PeptideCutter tool was used^41^.

### Circular dichroism spectroscopy

Far UV circular dichroism (CD) spectra of proteins and DRPs (concentration of 250 μg/ml in 6.5 mM Tris-HCl (pH 8) and in 6.5 mM Tris-HCl containing 63 mM hydrochloric acid (pH 1.2)) were recorded on a JASCO J-815 spectrometer (JASCO, Tokyo, Japan) at 37 °C at a spectral resolution of 0.5 nm at a scan rate of 100 nm/min over the wavelength range from 190–260 nm. Each spectrum was acquired sixteen times and the results were averaged. The experiments were performed in duplicate.

### De-novo modeling and molecular dynamic simulations

Sequences of two isoforms of Ara h 2 were obtained from UniProt (www.uniprot.org, identifiers Q6PSU2-1 and Q6PSU2-2). The missing regions in the Ara h 2.01 partial crystal structure (PDB code 3OB4) were built using Rosetta all-atom de-novo loop modeling procedure^42^, more specifically fragment-based loop modeling using kinematic closure (KIC)^43^. A total of 10 000 modeled structures were generated. More information about conformational space of missing regions was obtained by clustering the modeled structures by structural similarity. The lowest energy representative of the most populated cluster was taken for MD simulation.

The quality of obtained models were checked with programs ERRAT^44^ and PROCHECK^45^. Initial structures of the proteins for molecular dynamic simulations were obtained from Rosetta de novo modeling for Ara h 2.01 and Ara h 2.02 isoforms and from PDB (PDB code 1W2Q, model #1) for Ara h 6 protein. Structures of DRPs were obtained by deleting the trypsin cleaved amino-acids from the protein structure: for Ara h 2.02 peptide d from Fig. 3a, for Ara h 6 peptide d from Fig. 3c. The protonation state of each titratable amino-acid was estimated using H++ program^46^. Molecular dynamics (MD) calculations were performed with NAMD 2.9 program^47^ using the CHARMM27 force field^48^. Starting structures were solvated in a rectangular periodic box of TIP3P water molecules, with Na^+^ and Cl^−^ ions added to counter the total charge of the protein. Next, the system was subjected to extensive six-step equilibration protocol^49^, followed by 10 ns of NPT dynamics.

After equilibration, system was set to production run in NPT dynamics using the Langevin piston pressure control at 310 K and 1.01325 bar. Periodic boundary conditions and the Particle Mesh Ewald (PME) method were applied for a complete electrostatic calculation. The cut-off for non-bonded interactions was set to 9 Å, and switching function at 8 Å. Production phase was carried out with time step of 2 fs.

Root Mean Square Deviation (RMSD), Root Mean Square Fluctuations (RMSF) and Radius of gyration (Rg) were calculated in program VMD^50^ (version 1.9.2). For RMSF calculations a window of 20 frames width moving over trajectory was used. A STRIDE algorithm^51^ was used for analysing secondary structure changes over simulation time. Molecular graphics were created with the UCSF Chimera package^52^ (version 1.10.2). All calculations were done on PARADOX computer cluster.

### 2-DE and immunoblotting of digestion-resistant peptides

DRPs from individual or a mix of conglutins were diluted in rehydration buffer (6 M urea, 2 M thiourea, 2% CHAPS, 0.5% IPG buffer 3–10NL, 0.002% bromophenol blue and 50 mM DTT). Separation of peptides on the first dimension was carried out on an Ettan-IPGphor system (GE Healthcare, Uppsala, Sweden) according to manufacturer’s instruction. The samples (18.75 μg) were loaded on 13 or 7 cm immobilized pH gradient strips pH 3–10NL (GE Healthcare). After isoelectrofocusing strips were incubated for 15 minutes in 2D equilibration buffer (6 M urea, 75 mM Tris HCl pH 8.8, 29.3% glycerol, 2% SDS, 0.002% bromophenol blue) containing 10 mg/ml of DTT, and after that in equilibration buffer containing 25 mg/ml iodoacetamide, for 15 minutes in the dark. The second dimension was carried out on 16% gels. Imaging of the protein spots was performed with a Typhoon 7000 series laser scanner coupled with the Image 2D Master Platinum 7.0 software (GE Healthcare). In addition, DRPs from the conglutin mix were separated on 2-DE and transferred onto polyvinylidene fluoride (PVDF) membranes (Bio Rad). The membranes were blocked with 1% BSA in 20 mM Tris containing 0.9% NaCl pH 7.4 containing 0.05% Tween 20 (TBS-T), for 3 h at room temperature (RT). Subsequently, membrane was incubated overnight at 4 °C with 1:5 diluted serum pool from patients with proven peanut allergy. The serum pool was made from sera of seven peanut allergic patients (range and mean of total peanut-specific IgE: 53–787 kU/L and 254 kU/L respectively; range and mean of Ara h 2-specific IgE: 27–317 kU/L and 117 kU/L, respectively). Bound IgE was detected with mouse anti-human IgE antibody conjugated with alkaline phosphatase, diluted 1:2000 (Sigma-Aldrich), by incubation for 1 h at RT. The binding patterns were visualized with a substrate solution consisting of 1.5 mg 5-bromo-4-chloro-3′-indolyphosphate (BCIP) and 3 mg nitro-blue tetrazolium (NBT) in 10 mL of 100 mM Tris, containing 150 mM NaCl, and 5 mM MgCl_2_, pH 9.6.

### IgE binding properties of digestion-resistant peptides

The IgE binding properties of the native allergens and DRPs were analysed using an inhibition ELISA. The microtiter plates (NUNC MaxiSorp, Nunc A/S Plastfabrikation, Roskilde, Denmark) were coated with 100 μl of 5 μg/ml native proteins per well in coating buffer (15 mM Na_2_CO_3_, 35 mM NaHCO_3_ pH 9.6) and incubated overnight at 4 °C. The remaining binding sites were blocked with 1% BSA in TBS-T for 1 h at RT. A dilution series of the samples in 0.1% BSA in TBS-T was incubated with the serum pool (the same serum pool as was used for immunoblotting, diluted 100-fold), directly on the plate for 1 h at RT. The concentration range of allergens was 0.004–10 μg/ml. Detection was performed by incubation with 100 μl mouse-anti-human IgE monoclonal antibody (Abcam, UK, 2000 times diluted in TBS-T containing 0.1% BSA) conjugated to horseradish peroxidase for 1 h at RT and 3,3′,5,5′-tetramethylbenzidine (TMB) as substrate. The concentration needed to inhibit 50% of the signal was used to compare IgE-binding potencies of samples (IC_50_ value).

## Additional Information

**How to cite this article**: Apostolovic, D. *et al*. Conformational stability of digestion-resistant peptides of peanut conglutins reveals the molecular basis of their allergenicity. *Sci. Rep.* **6**, 29249; doi: 10.1038/srep29249 (2016).

## Supplementary Material

Supplementary Information
