# Supplementary material for: Conformational stability of digestion-resistant peptides of peanut conglutins reveals the molecular basis of their allergenicity
Source: Sci Rep. 2016 Jul 5;6:29249. doi: 10.1038/srep29249 (PMC4932508; doi:10.1038/srep29249)
Supplement: Supplementary Information [file srep29249-s1.doc]

**Supplementary Information**

**Title: Conformational stability of digestion-resistant peptides of peanut conglutins reveals the molecular basis of their allergenicity**

**Authors:** Danijela Apostolovic^1^, Dragana Stanic-Vucinic^1^, Harmen H. J. de Jongh^2^, Govardus A.H. de Jong^3^, Jelena Mihailovic^1^, Jelena Radosavljevic^1^, Milica Radibratovic^4^, Julie A. Nordlee^5^, Joseph L. Baumert^5^, Milos Milcic^1^, Steve L. Taylor^5^, Nuria Garrido Clua^2^, Tanja Cirkovic Velickovic^1*^, Stef J. Koppelman^5*^

**Affiliations:**

^1^ Center of Excellence for Molecular Food Sciences, Faculty of Chemistry, University of Belgrade, Studentski trg 16, 11000 Belgrade, Serbia;

^2^ TI Food and Nutrition, P.O. Box 557, 6700 AN Wageningen, the Netherlands;

^3^ TNO, Utrechtseweg 48, 3704 HE, Zeist, the Netherlands;

^4^Institute of Chemistry, Technology and Metallurgy - Center of Chemistry, Njegoseva 12, Belgrade, Serbia;

^5^ Food Allergy Research and Resource Program, University of Nebraska, 279 Food Innovation Center, Lincoln, Nebraska 68588-6207, USA.

^*^ equally contributing authors

**Correspondence:**

Prof. dr Tanja Cirkovic Velickovic

Center of Exellence for Molecular Food Sciences, University of Belgrade – Faculty of Chemistry, Studentski trg 16, 11000 Belgrade, Serbia; [tcirkov@chem.bg.ac.rs](mailto:tcirkov@chem.bg.ac.rs)

Prof. dr Stef J. Koppelman

Food Allergy Research and Resource Program, University of Nebraska, 279 Food Innovation Center, Lincoln, Nebraska 68588-6207, USA; [stefkoppelman@zonnet.nl](mailto:stefkoppelman@zonnet.nl)

**Table S1. Masses of identified digestion-resistant peptides from conglutin isoforms**

| **DRP** | | **Sequence**  **mass** | | **pI** | **Modifications** | | | | | | | | | **Calculated mass** | | **Experimental mass** | |
| --- | --- | --- | --- | --- | --- | --- | --- | --- | --- | --- | --- | --- | --- | --- | --- | --- | --- |
|  | | | | | | H_2_O ^a)^ | *Disulfide bond* | | *Alkyl group* | *Proline hydroxylation* | | *Sequence conflict* | | |  | |  |
| **Ara h 2.02 non-reduced** | | | | | | | | | | | | | | | | | |
| a | 17993.69 | | 5.36 | | +18 | | | -8 | 0 | | +48 | | 0 | | 18051.69 | 18051.33 | |
| b | 17559.24 | | 5.35 | | +18 | | | -8 | 0 | | +48 | | 0 | | 17617.24 | 17617.14 | |
| c | 16659.28 | | 5.34 | | +18 | | | -8 | 0 | | +32 | | 0 | | 16701.28 | 16700.72 | |
| d | 16224.82 | | 5.33 | | +18 | | | -8 | 0 | | +32 | | 0 | | 16266.82 | 16266.55 | |
| e | 16068.64 | | 5.1 | | +18 | | | -8 | 0 | | +32 | | 0 | | 16110.64 | 16110.44 | |
| f | 3541.67 | | 4.17 | | 0 | | | 0 | 0 | | +48 | | 0 | | 3589.67 | 3589.57 | |
| g | 3030.04 | | 3.79 | | 0 | | | 0 | 0 | | +32 | | 0 | | 3062.04 | 3061.28 | |
| h | 4364.46 | | 4.01 | | 0 | | | 0 | 0 | | +48 | | 0 | | 4412.46 | 4411.90 | |
| **Ara h 2.02 reduced and alkylated** | | | | | | | | | | | | | | | | | |
| i | 9401.39 | | 5.2 | | 0 | | | 0 | +342 | | 0 | | 0 | | 9743.39 | 9743.58 | |
| i | 9401.39 | | 5.2 | | 0 | | | -2 | +285 | | 0 | | 0 | | 9684.39 | 9684.58 | |
| i | 9401.39 | | 5.2 | | 0 | | | 0 | +228 | | 0 | | 0 | | 9629.39 | 9629.53 | |
| j | 8966.94 | | 5.18 | | 0 | | | 0 | +342 | | 0 | | 0 | | 9308.94 | 9308.38 | |
| j | 8966.94 | | 5.18 | | 0 | | | 0 | +285 | | 0 | | 0 | | 9251.94 | 9252.88 | |
| j | 8966.94 | | 5.18 | | 0 | | | 0 | +228 | | 0 | | 0 | | 9194.94 | 9194.35 | |
| j | 8966.94 | | 5.18 | | 0 | | | -2 | +171 | | 0 | | 0 | | 9135.94 | 9135.31 | |
| k | 8610.31 | | 5.64 | | 0 | | | 0 | +114 | | +48 | | 0 | | 8772.31 | 8772.16 | |
| k | 8610.31 | | 5.64 | | 0 | | | 0 | +57 | | +48 | | 0 | | 8715.31 | 8714.16 | |
| l | 8454.13 | | 5.05 | | 0 | | | 0 | +114 | | +48 | | 0 | | 8616.13 | 8615.10 | |
| m | 7275.9 | | 5.61 | | 0 | | | 0 | +114 | | +32 | | 0 | | 7421.90 | 7421.57 | |
| **Ara h 2.01 non-reduced** | | | | | | | | | | | | | | | | | |
| a | 16579.23 | | 5.67 | | 0 | | | -8 | 0 | | +32 | | +92 | | 16695.23 | 16695.72 | |
| b | 16423.04 | | 5.33 | | +18 | | | -8 | 0 | | +32 | | +92 | | 16557.04 | 16555.50 | |
| b | 16423.04 | | 5.33 | | 0 | | | -8 | 0 | | +32 | | +14 | | 16461.04 | 16462.54 | |
| c | 16416.06 | | 5.67 | | +18 | | | -8 | 0 | | +32 | | 0 | | 16458.06 | 16456.54 | |
| d | 16259.87 | | 5.1 | | +18 | | | -8 | 0 | | +32 | | +58 | | 16359.87 | 16359.54 | |
| e | 16103.68 | | 5.1 | | +18 | | | -8 | 0 | | +32 | | +58 | | 16203.68 | 16202.52 | |
| f | 1971.03 | | 3.59 | | 0 | | | 0 | 0 | | +32 | | 0 | | 2003.03 | 2002.83 | |
| **Ara h 2.01 reduced and alkylated** | | | | | | | | | | | | | | | | | |
| g | 9238.21 | | 5.73 | | 0 | | | 0 | +171 | | 0 | | 0 | | 9409.21 | 9409.40 | |
| g | 9238.21 | | 5.73 | | 0 | | | 0 | +114 | | 0 | | 0 | | 9352.21 | 9352.31 | |
| g | 9238.21 | | 5.73 | | 0 | | | 0 | +57 | | 0 | | 0 | | 9295.21 | 9295.29 | |
| g | 9238.21 | | 5.73 | | 0 | | | 0 | 0 | | 0 | | 0 | | 9238.21 | 9238.31 | |
| h | 7195.86 | | 7.02 | | 0 | | | 0 | +171 | | +32 | | +14 | | 7412.86 | 7413.49 | |
| i | 7039.67 | | 5.59 | | 0 | | | 0 | +171 | | +32 | | +14 | | 7256.67 | 7257.43 | |
| j | 2127.21 | | 4.17 | | 0 | | | 0 | 0 | | +32 | | 0 | | 2159.21 | 2158.94 | |
| **Ara h 6 non-reduced** | | | | | | | | | | | | | | | | | |
| a | 14116.86 | | 4.83 | | +18 | | -10 | | 0 | | 0 | | 0 | | 14124.86 | 14125.29 | |
| b | 13903.62 | | 4.68 | | +18 | | -10 | | 0 | | 0 | | 0 | | 13911.62 | 13911.17 | |
| c | 13772.49 | | 4.68 | | +18 | | -10 | | 0 | | 0 | | 0 | | 13780.49 | 13780.12 | |
| d | 13559.25 | | 4.55 | | +18 | | -10 | | 0 | | 0 | | 0 | | 13567.25 | 13567.02 | |
| **Ara h 6 reduced and alkylated** | | | | | | | | | | | | | | | | | |
| e | 9148.37 | | 4.78 | | 0 | | -2 | | 285 | | 0 | | 0 | | 9431.37 | 9432.23 | |
| e | 9148.37 | | 4.78 | | 0 | | -2 | | 228 | | 0 | | 0 | | 9374.37 | 9374.24 | |
| e | 9148.37 | | 4.78 | | 0 | | -2 | | 171 | | 0 | | 0 | | 9317.17 | 9317.22 | |
| e | 9148.37 | | 4.78 | | 0 | | -2 | | 114 | | 0 | | 0 | | 9260.37 | 9260.19 | |
| e | 9148.37 | | 4.78 | | 0 | | -2 | | 57 | | 0 | | 0 | | 9203.37 | 9203.17 | |
| f | 5330.88 | | 5.56 | | 0 | | 0 | | 171 | | 0 | | 0 | | 5501.88 | 5501.62 | |
| f | 5330.88 | | 4.9 | | 0 | | 0 | | 114 | | 0 | | 0 | | 5444.88 | 5444.59 | |
| g | 5117.64 | | 4.9 | | 0 | | 0 | | 171 | | 0 | | 0 | | 5288.64 | 5288.50 | |
| g | 5117.64 | | 4.9 | | 0 | | 0 | | 114 | | 0 | | 0 | | 5231.64 | 5231.47 | |
| h | 4986.51 | | 4.9 | | 0 | | 0 | | 228 | | 0 | | 0 | | 5214.51 | 5214.47 | |
| h | 4986.51 | | 4.9 | | 0 | | 0 | | 171 | | 0 | | 0 | | 5157.51 | 5157.45 | |
| h | 4986.51 | | 4.9 | | 0 | | 0 | | 114 | | 0 | | 0 | | 5100.51 | 5100.43 | |
| i | 4773.27 | | 4.55 | | 0 | | 0 | | 171 | | 0 | | 0 | | 4944.27 | 4943.47 | |
| i | 4773.27 | | 4.55 | | 0 | | 0 | | 114 | | 0 | | 0 | | 4887.27 | 4886.29 | |
| i | 4773.27 | | 4.55 | | 0 | | 0 | | 57 | | 0 | | 0 | | 4830.27 | 4829.27 | |

^a)^ Internal peptide bond hydrolysis

**Supplementary Figure 1. Time course of trypsin digestion of conglutin isoforms as analysed by SDS-PAGE.**


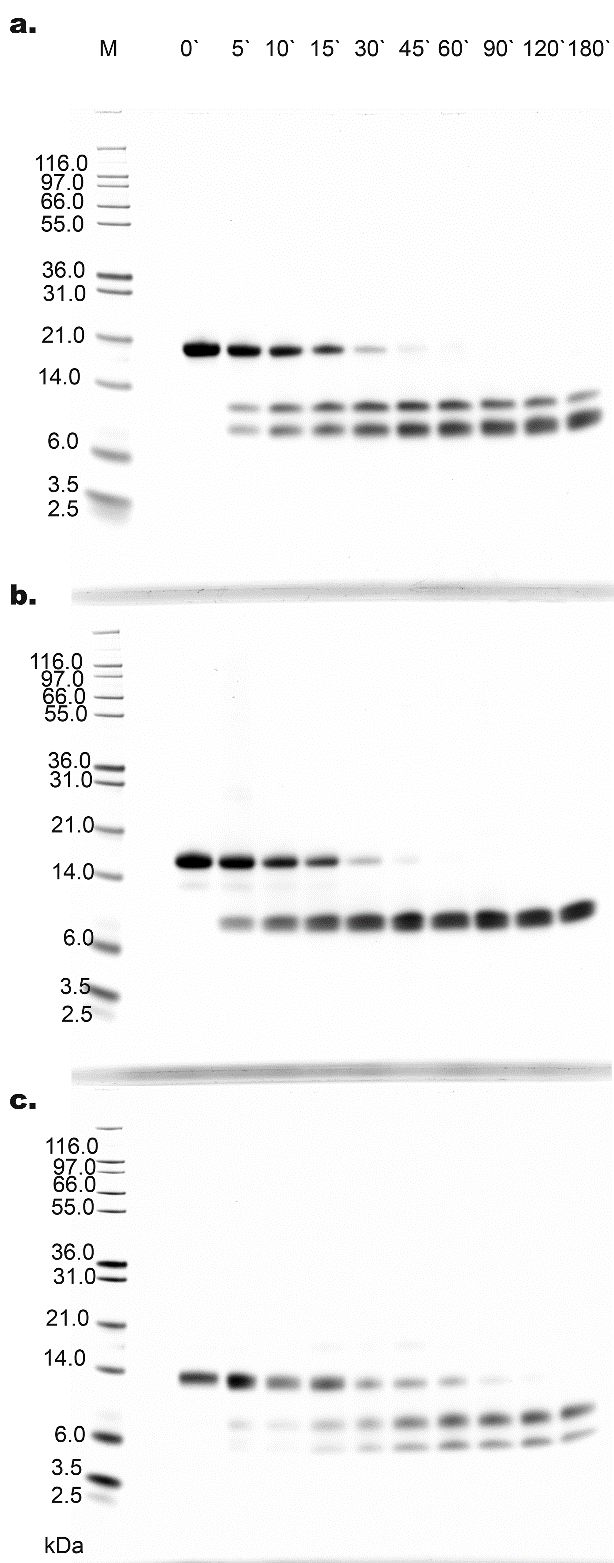


Incubation times (in minutes) are indicated at the top of each pane. Panel a: Ara h 2.02; Panel b: Ara h 2.01; Panel c: Ara h 6. M: Molecular markers. For all panels reducing conditions were applied.

**Supplementary Figure 2. 2-DE analysis of DRPs from conglutin isoforms**


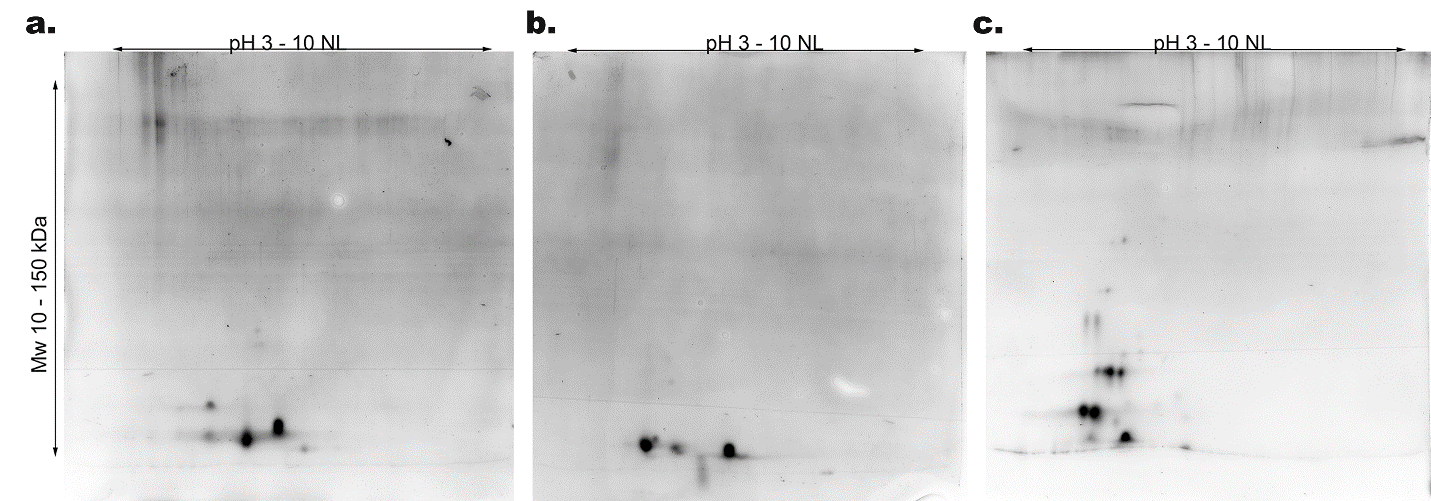


Panel a: DRPs of Ara h 2.02; Panel b: DRPs of Ara h 2.01; Panel c: DRPs of Ara h 6

**Supplementary Figure 3. Sequence alignment and2D topology diagrams of conglutin isoforms**

**
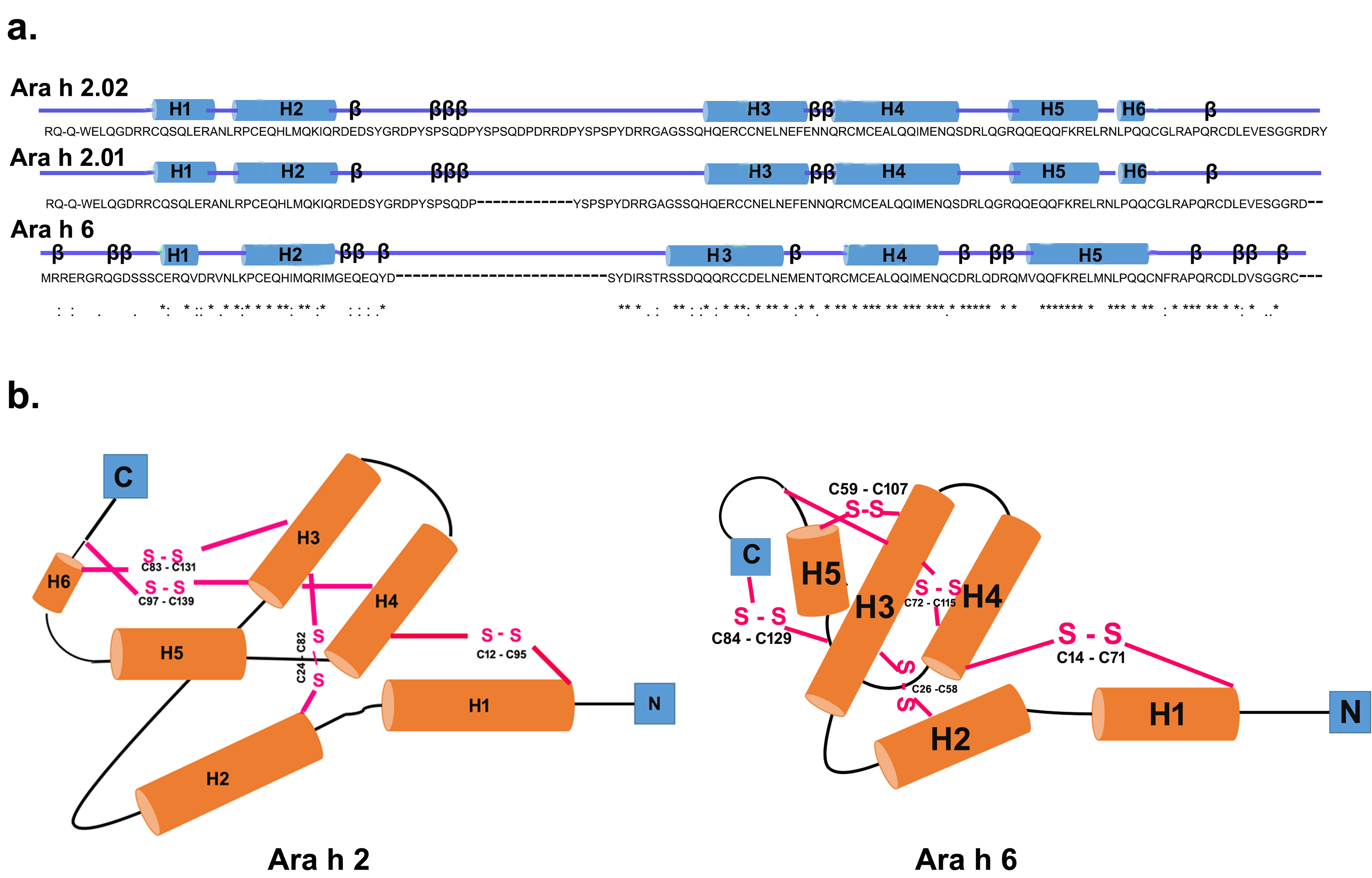
**

**Panel a** – sequence alignment of conglutin isoforms with assign secondary structures. Symbols underneath the alignment indicate: positions which have a single, fully conserved residue (*), conservation between groups of strongly similar properties (**:**) and weakly similar properties (**.**) Blue cylinder represent helix, and β strands and turns based on PDB sum for PDB codes: 3OB4 (Ara h 2) and 1w2q (Ara h 6).

**Panel b** - topology diagram of Ara h 2.02 and Ara h 6 with assigned disulphide bridges. Orange cylinder represent helix; s-s connection for specific cysteine residue into the disulphide bridge; N in blue square represent N terminus of protein; C in blue square represent C terminus of protein.

**Supplementary Figure 4. RMSD variation of conglutin isoforms with DRP**


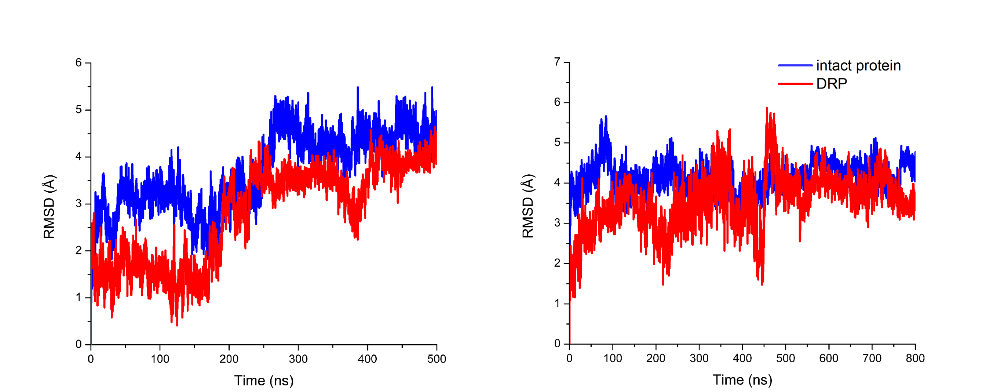


Panel a: Ara h 2.02; Panel b: Ara h 6.

**Supplementary Figure 5. RG variation of conglutin isoforms with DRP**


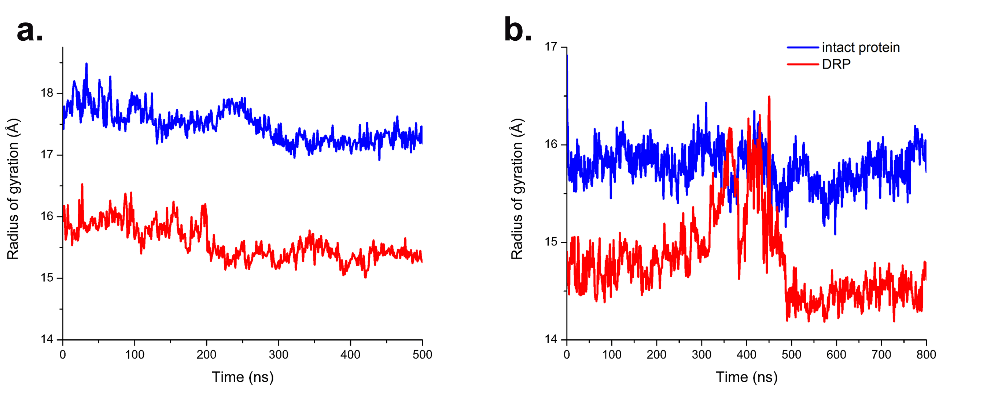


Panel a: Ara h 2.02; Panel b: Ara h 6.

**Supplementary Figure 6. Alpha helix in DRPs calculated according to STRIDE algorithm.**

**
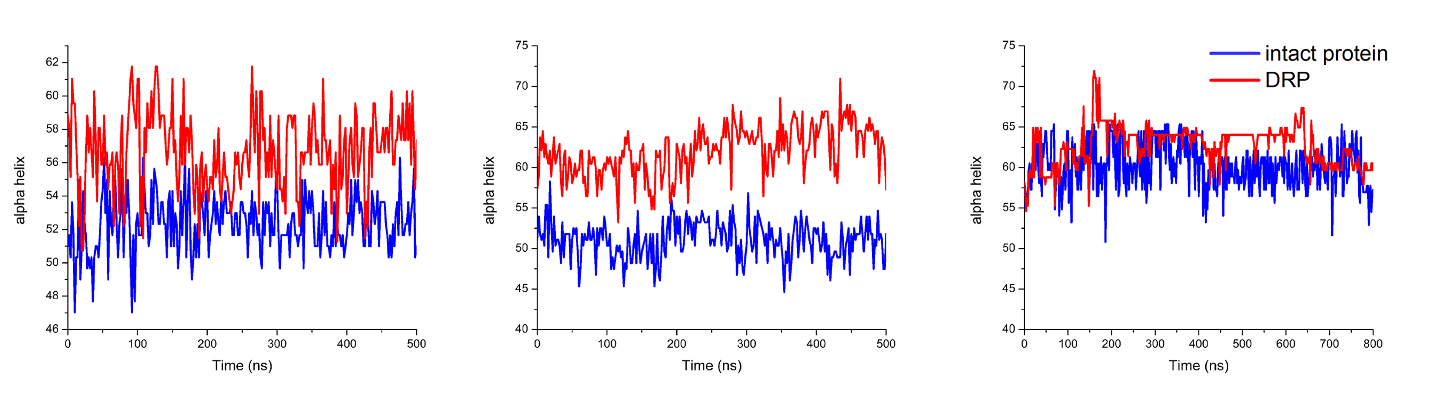
**

alpha helix in intact conglutins (blue) and DRPs red). Panel a: Ara h 2.02. Panel b: Ara h 2.01. Panel c: Ara h 6.
